# Dogs’ social susceptibility is differentially affected by various dog–Human interactions. A study on family dogs, former shelter dogs and therapy dogs

**DOI:** 10.1371/journal.pone.0300889

**Published:** 2024-03-21

**Authors:** Anna Kis, Katinka Tóth, Lívia Langner, József Topál

**Affiliations:** 1 Institute of Cognitive Neuroscience and Psychology, Budapest, Hungary; 2 ELTE-HunRen NAP Comparative Ethology Research Group, Budapest, Hungary; Universidade do Porto Instituto de Biologia Molecular e Celular, PORTUGAL

## Abstract

When pre-treated with social stimuli prior to testing, dogs are more susceptible to human influence in a food preference task. This means, after a positive social interaction they are more willing to choose the smaller amount of food indicated by the human, as opposed to their baseline preference for the bigger amount. In the current study we investigate if and how various forms of social interaction modulate choices in the same social susceptibility task, testing dogs with varying early life history (pet dogs, therapy dogs, former shelter dogs). In line with previous studies, dogs in general were found to be susceptible to human influence as reflected in the reduced number of “bigger” choices in the human influence, compared to baseline, trials. This was true not only for pet dogs with a normal life history, but also for dogs adopted from a shelter. Therapy dogs, however, did not uniformly change their preference for the bigger quantity of food in the human influence trials; they only did so if prior to testing they had been pre-treated with social stimuli by their owner (but not by a stranger). Pet dogs were also more influenced after pre-treatment with social stimuli by their owner compared to ignoring and separation; however after pre-treatment by a stranger their behaviour did not differ from ignoring and separation. Former shelter dogs on the other hand were equally influenced regardless of pre-treatment by owner versus stranger. In summary these results show that dogs’ social susceptibility is modulated by both interactions immediately preceding the test as well as by long term social experiences.

## Introduction

Due to their special domestication history, dogs show a range of human-like social behaviours [1]. Many of these socio-cognitive skills are obviously advantageous when dogs navigate the human environment: e.g. the ability to follow human gestures [2] and to correctly differentiate dog directed speech [3]. There are, however, also results showing that due to their susceptibility to human cues, dogs make counterproductive choices in a range of experimental situations. For example dogs choose an empty container to which an experimenter points, ignoring olfactory cues, although they choose the baited container when only olfactory cues are available [4]. Dogs are also prone to the so-called A-not-B error in hide-and-seek tasks, that is they will erroneously search at if the hiding is accompanied by human ostensive cues, even if they witness the hiding at the other location [5]. Dogs are more likely to select a visibly non-baited container after a communicative demonstration by the experimenter compared to a non-communicative demonstration, especially if the experimenter remains in the dog’s presence [6]. Dogs that typically choose the larger of two food quantities when alone, can be induced to lose that preference when their owner vocally and behaviourally shows an interest for the smaller food quantity [7]. Together these results show that if human ostensive cues are presented concurrently during a behavioural test, they have a substantial influence on dogs’ performance.

Human studies have shown that in addition to concurrent social cues, pre-treatment with positive social interactions alone can also change subjects’ social behaviour such as monetary sacrifice among strangers [8] or emotion and trustworthiness judgement of faces [9]. Recently similar results were found in dogs using a social susceptibility paradigm, which measures in a food preference task how much dogs are willing to conform to the experimenter’s counterproductive choice of the smaller quantity [7]. A 10-minute long social interaction with the owner, in contrast to a socially ignoring pre-treatment (owner present, but ignoring the dog), increased the number of counterproductive choices in the human influence compared to the baseline trials. This effect is most likely driven by dogs’ endogenous oxytocin increase, as the social stimulation versus ignoring results paralleled the effect of intranasal oxytocin (contrasted to placebo) administration [10]. There is also independent physiological evidence showing elevated oxytocin levels after the social treatment used in that study [11].

Research in dogs has shown that peripheral oxytocin levels increase following a positive social interaction with both the owner [12, 13] as well as a stranger human [14, 15] (although see others [16, 17] for no oxytocin increase after dog-human social interaction). It is important to notice that the relationship between individuals who interact can substantially influence the endogenous changes it drives. The first study to draw attention to this fact in the oxytocin field was conducted on chimpanzees, and it has been shown [18] that oxytocin levels were higher after grooming with bond partners compared with non-bond partners. Dogs have a special attachment bond to their owners [19] and thus across a range of tasks they react differentially at the behavioural level depending on whether they interact with their owner or a stranger (e.g. in an attention task [20], everyday interactions [21], emotion recognition [22]). Recently it has been shown [23] in a cuddle test, where during 5 minutes the animals were free to approach the fence to be petted by a human partner, that the time pet dogs spent in physical contact with their owners, but not with a familiar person is positively associated with urinary oxytocin concentrations.

The above cited food choice study [10] showing an effect of positive social interaction on social susceptibility has used the owner as the interacting partner. Here in Study 1. we aim to replicate those conditions (social interaction with the owner and social ignoring) and complement them with a condition using social interaction with a stranger. While social ignoring seems to serve as a good parallel to the neutral placebo condition, oxytocin levels can be decreased in stressful situations (via increasing cortisol [24], although see Ogit et al. [25] for a negative result). Thus, as a fourth condition, the present study will also use a social isolation pre-treatment.

In addition to social interactions immediately preceding the behavioural test (experimental pre-treatments) the life history of the subjects, in general, might also influence how they perform. A considerable proportion of dogs are adopted from a shelter and thus have experienced some form of social trauma and isolation. Most of the literature on (former) shelter dogs focuses on behavioural problems post-adoption [26] and the potential to predict such problems while the dogs are still in the shelter [27]. However, some have also reported deficits in the way they interact with humans (reviewed in [28]). Inferior performance in pointing following has been shown by several studies [29–31], although others have found no such effects [32]. Shelter dogs also differ from pet dogs in sustaining eye-contact [33]. Another group of crucial interest are therapy dogs, trained to work in a setting where they engage in positive dog-human interactions [34]. Recent research into therapy dog welfare suggests that animal assisted interventions are not stressful or even are relaxing for the dogs [35] (although others still raise potential welfare considerations [36]). Study 2. of the current paper will focus on both former shelter dogs and therapy dogs in addition to pet dogs.

### Ethical statement

This research was approved by the National Animal Experimentation Ethics Committee (Ref No. PE/EA/55-4/2019). All dog owners provided informed written consent for their dogs to participate in the study; dog owners themselves did not participate as subjects in the study. Research was done in accordance with the Hungarian regulations on animal experimentation and the guidelines for the use of animals in research described by the Association for the Study Animal Behaviour (ASAB). The authors did not have access to personal data of the owners during the study, all statistical analyses focused solely on dogs.

## Study 1

### Methods

#### Subjects

Adult (over 1 year of age) pet dogs were tested with all owners volunteering their dog to participating in the experiment. The admission criteria was for dogs to be older than 1 year and have been born at a breeder (not acquired e.g. from a shelter). We further excluded dogs that showed a consistent side preference (choosing either left or right on 100% of the trials in both test phases, N = 15) and if unwilling to take the food reward (N = 3). Thus the total number of subjects included was N = 64 dogs of various breeds and ages (mean ±SD = 4.25 ±2.89 years), 26 males, 38 females.

#### Pre-treatment

Subjects received a 10-minute-long pre-treatment according to the treatment group to which they were randomly assigned to (N = 16 dogs/group). These included (1) social interaction with the owner, (2) social interaction with a stranger, (3) social ignoring, and (4) social separation.

Social interaction with the owner included 10 minutes of positive social interaction where the owner was instructed to remain seated at a fixed position (on a chair) with the experimenter similarly seated at a 2 m distance (not interacting with either the dog or the owner). The owner was instructed to talk to the dog as they naturally would (using dog-directed speech), and to pet the dog looking into their eyes as much as possible. In addition for the second half of the pre-treatment (5 minutes in duration) the owner was allowed to use a toy (provided by the experimenter) to get the dog’s attention and lure it closer as well as to instruct it to make eye-contact. All owners used the toy, but the duration and intensity of their movements varied as they were allowed to behave as they normally would, so that the interaction was natural for the dogs. We had previously confirmed that this exact interaction protocol increases dogs’ peripheral oxytocin levels compared to baseline [11].

Social interaction with a stranger was carried out following the above protocol with the difference that this time it was a stranger (always from the same gender as the dog’s owner and not the same person that would act as the Experimenter carrying out the Food Preference test) was interacting with the dog, while the owner was sitting on the other chair without intervening.

The Social ignoring condition was carried out with the same setup as above with both the Owner and the experimenter being seated on a chair, but this time, none of them was interacting with the dog. Instead the dog received a toy at the beginning of the 10 minutes, so that it could engage in solitary play, and the experimenter talked to the owner in the form of questions and answers following a personality questionnaire.

For the Social separation condition the dog was left alone in the room for the 10-minute-long duration of the pre-treatment with both the owner and the experimenter waiting outside the room. The same toys were provided for the dog as in the other conditions. The owner and the experimenter were monitoring the dog from outside in order to make sure that the treatment did not cause an unacceptable level of stress in the dog. The owners were aware that they could terminate the experiment and withdraw the dog from the project at any time and without having to give a justification; but none of the owners felt that the separation caused too much stress to the dog.

#### Behavioural test

The pre-treatments were immediately followed by a behavioural test aimed to test social susceptibility via measuring food preference choice [7, 37]. The task consisted of (1) a baseline phase (free choice between bigger and smaller quantities– 1 vs. 8 food pellets) and (2) a human influence phase (choice between the same bigger and smaller quantities, but this time with the experimenter clearly showing a preference for the smaller one). The test was made up of 6 baseline trials followed by 6 human influence trials, which were carried out consecutively without any break between trials.

Baseline phase. The owner was instructed to hold the dog on leash while seated on a chair at a predetermined position. The experimenter approached the dog from the front and showed two plates to the dog (containing 1 or 8 food pellets respectively in a way that is clearly visible to the dog). Then the experimenter went two steps backward and placed the plates on the left and right side on the floor (same side as they had been shown to the dog), while taking care never to look at the dog or at either of the plates. The experimenter then stood up looking at the floor, and the owner released the dog that was free to make a choice between the two plates. There was no pre-set time restriction for dogs to make a choice, and all subjects immediately went for one of the containers, taking a maximum of 10 seconds in their choice. The dog was allowed to eat the content of the first plate it visited, but the experimenter immediately removed the non-chosen plate.

Human preference phase. The dog and the owner were sitting in the same position as in the baseline condition. The experimenter approached the dog in the same way, and placed the two plates with the bigger (8 pellets) and smaller (1 pellet) food quantities on the floor as in the baseline condition. The experimenter then approached the plate containing the smaller food quantity, picked up the piece of food and with an enthusiastic tone of voice, said: “Mmm, yummy, this is delicious!”, while all the time taking care never to look at the dog (as such ostensive cues would cause a ceiling effect in dogs choices). The experimenter then placed the piece of food back on the plate and stepped back to the middle position behind the plates looking at the floor. At this point the owner released the dog, that was allowed to make a choice as in the baseline condition.

#### Analysis

Subjects’ choice was scored in all trials throughout the 6 Baseline and 6 Human influence conditions. A trial received score 1 if the dog chose the plate with bigger quantity of food (8 pellets), and score 0 if the plate with smaller quantity (0 pellets) was chosen.

Choice scores for all 12 trials were entered into a Generalized Linear Mixed Model (binary logistic, including subject ID and trial order as confounding variables). The tested variables of interest were the main effect of phase (Baseline vs. Human influence) and pre-treatments (Social interaction with the owner, Social interaction with a stranger, Social ignoring, Social separation) as well as the interaction of these two factors.

### Results

**Fig 1:**
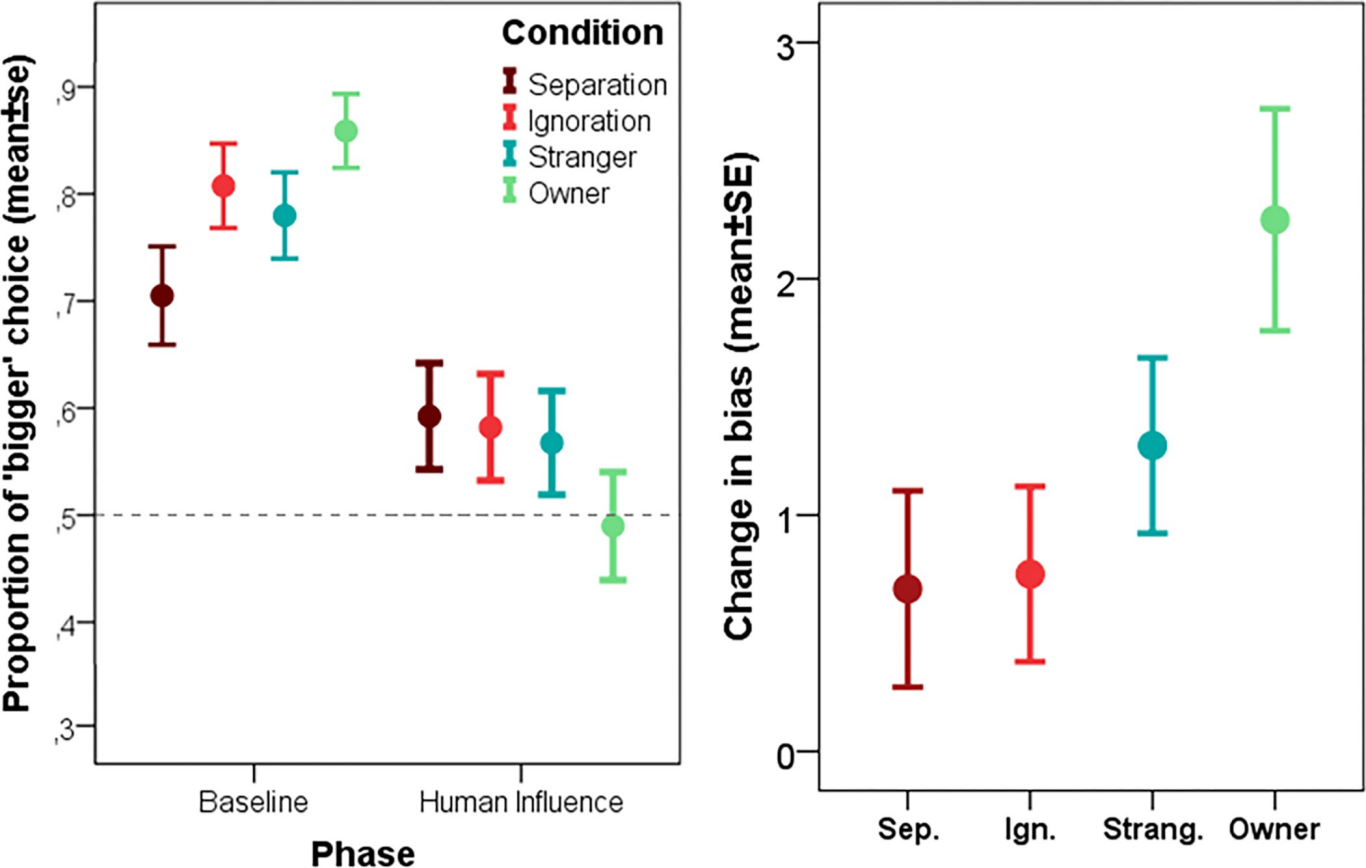
The differential reaction of dogs to human influence following the four different pre-treatments (Social separation, social ignoring; social interaction with a stranger, social interaction with the owner). **Fig 1/a** shows the proportion of “bigger” choices during the Baseline and the Human influence trials in the four different pre-treatment groups. **Fig 1/b** shows “change in bias” scores (difference between baseline and human influence trials) for the same four treatment groups.

Dogs were found to be susceptible to human influence (similarly to previous studies [7, 38, 39]) as they produced significantly less “bigger” choices when influenced by humans compared to baseline (main effect of phase: F = 50.821; p<0.001). As expected, pre-treatment conditions (Social interaction with the owner, by the stranger, Social ignoring, Social separation) did not have a main effect in themselves (all p>0.05), but the phase × condition interaction was significant (F = 3.180; p = 0.023) meaning that the pre-treatments differentially modulated subjects’ behaviour in the human influence phase (**Fig 1**). Post-hoc analysis showed that the change in dogs’ bias (from baseline to human influence) was greater in the owner pre-treatment condition compared to separation (p = 0.045) and social ignoring (p = 0.050), but it was not different from the stranger pre-treatment (p = 0.349). The ‘Social interaction with a stranger’ pre-treatment did not differ from the ‘Social separation’ and ‘Social ignoring’ conditions either (p>0.05) and the two control conditions were also non-different (p = 0.715).

## Study 2

### Background

Dogs with varying early life history and social experiences might react differently to interaction with humans. Thus in *Study 2* we recruited therapy dogs and former shelter dogs. Therapy dogs, similarly to pet dogs, have been acquired from a responsible breeder and now live in human families, but have received additional training and experience in therapy settings. Former shelter dogs currently live in human families, but they have previously experienced being relinquished to an animal shelter in their earlier lives.

### Methods

#### Subjects

Subjects with different early life history were selected to test the effect of human social stimulation (owner or stranger). Adult (over 1 year of age) dogs either having been acquired from a responsible breeder and additionally trained for therapy work (N = 27) or adopted from a shelter (N = 28) from various breeds and ages (mean ±SD = 3.42±2.53 years), 22 males, 34 females were tested. All owners volunteered to participate in the experiment, and there were no admission criteria (other than the specified early-life history and being older than 1 year), however we did exclude dogs that showed a consistent side preference (choosing either left or right on 100% of the trials in both test phases, N = 5). Both subject groups (therapy dogs and former shelter dogs) were divided into two pre-treatment groups (social stimulation with owner or stranger) in a 2×2 design so that the final sample consisted of N = 14 former shelter dogs pre-treated with social interaction with the owner and N = 14 former shelter dogs with social interaction with the experimenter as well as N = 14 therapy dogs with social interaction with the owner, and N = 13 therapy dogs with social interaction with the experimenter.

#### Pre-treatment

The pre-treatments in both groups (Social interaction with the owner or stranger) were carried out in the same way as described above in Study 1. They included 10 minutes of positive social interaction where the owner and the stranger were seated at a 2 m distance and one of them was instructed to talk to the dog as she/he naturally would (using dog-directed speech), petting the dog and looking into its eyes as much as possible. In addition, for the second half of the pre-treatment (last 5 minutes) the owner / the stranger was allowed to use a toy to facilitate interaction with the dog.

#### Food preference test

The behavioural test was identical to that of Study 1. There were 6 baseline trials (choice between bigger and smaller quantities– 1 vs. 8 food pellets) followed by 6 human influence trials (choice between the same bigger and smaller quantities, but this time with the experimenter clearly showing a preference for the smaller one).

#### Analysis

Subjects’ choice was again scored in all 12 trials with score 1 for choosing the bigger quantity of food (8 pellets), and score 0 for the smaller quantity (0 pellets).

Data from the two subject groups (therapy dogs and former shelter dogs) were analysed in two separate models in the same way as described above for the pet dogs (Generalized Linear Mixed Model, binary logistic) with the main effects of phase (baseline vs human influence) and condition (social pre-treatment with owner vs stranger) and the interaction of the two factors.

### Results

**Fig 2:**
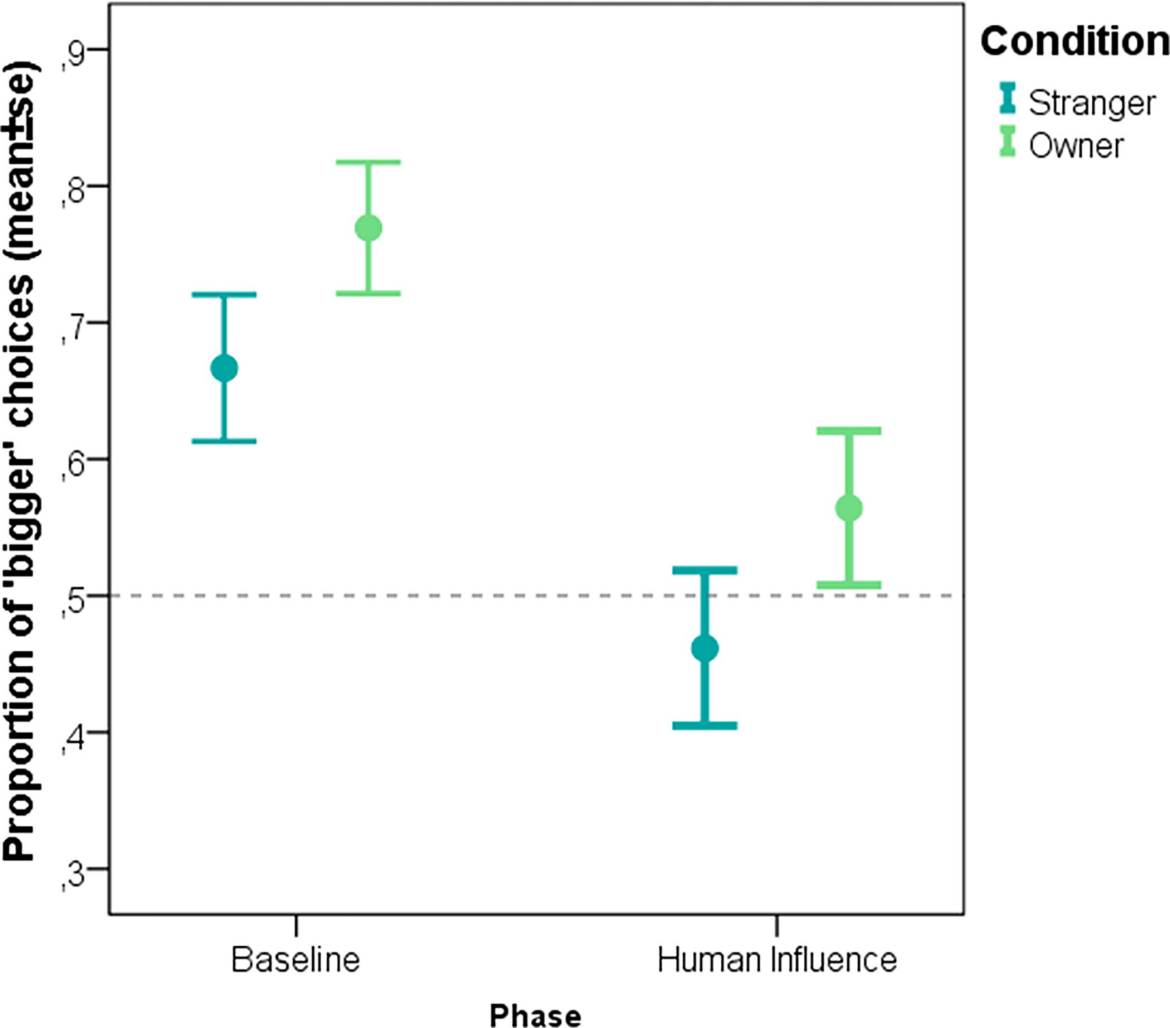
The proportion of former shelter dogs’ “bigger” choices during the baseline and the human influence trials in the two different pre-treatment groups (Social interaction with a stranger, social interaction with the owner).

Former shelter dogs were found to be influenced by human preference, that manifested in the main effect of phase (F = 14.086, p<0.001). There was no effect of social influence by owner vs stranger (pre-treatment main effect: F = 2.44, p = 0.12) nor by phase × condition interaction (F = 0.31, p = 0.58; **Fig 2**).

**Fig 3:**
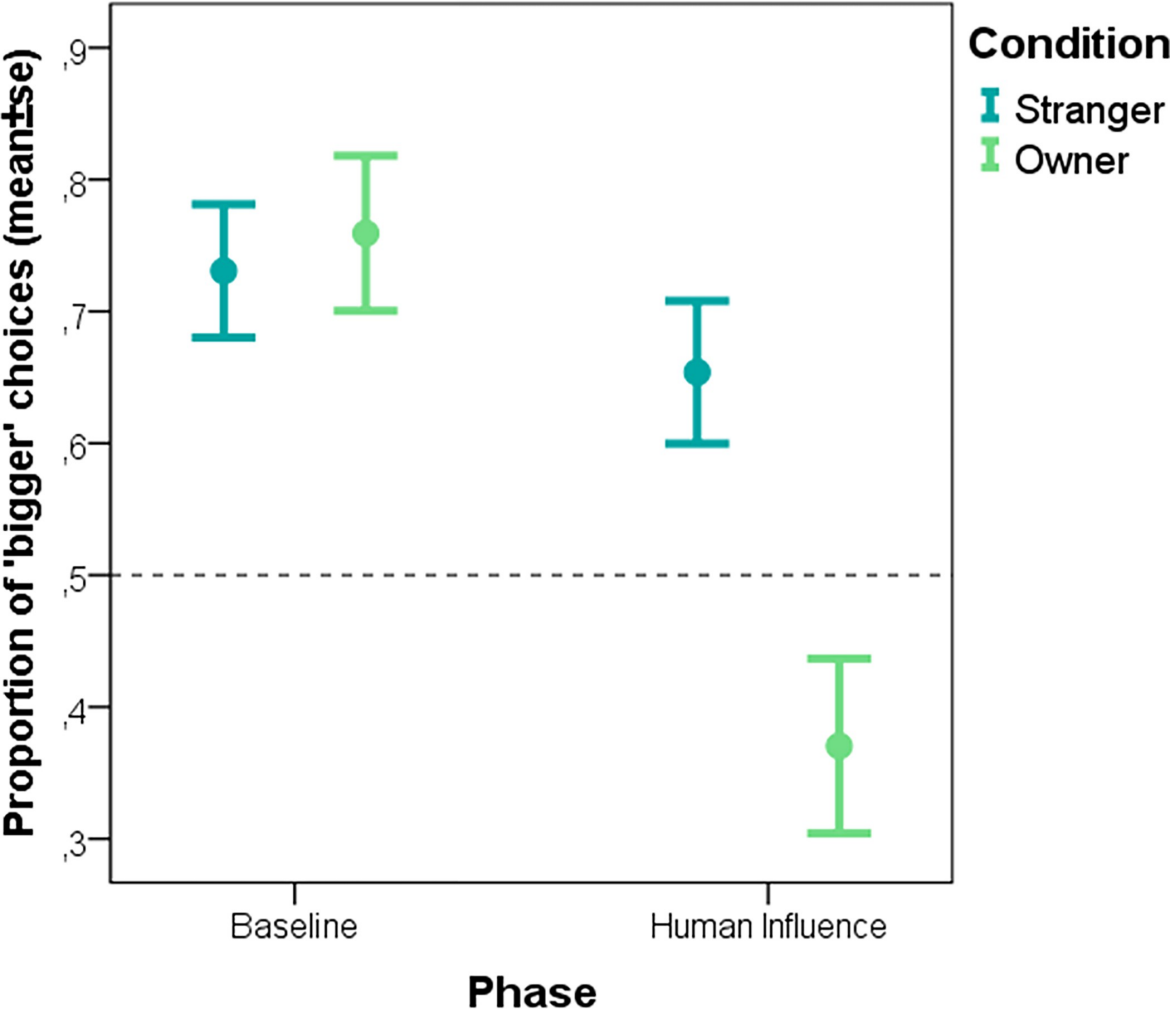
The proportion of therapy dogs’ “bigger” choices during the baseline and the human influence trials in the two different pre-treatment groups (Social interaction with a stranger, social interaction with the owner).

Therapy dogs, on the other hand, were not only influenced by phase (F = 12.813; p<0.001), but the phase × condition interaction also had a significant effect (F = 4.460; p = 0.036), showing that they followed the human’s preference more if pre-treated with social stimuli by the owner, and not the stranger (Fig 3). The main effect of condition was not significant (p>0.05).

For visual comparison, the “change in bias” scores of dogs adopted from a shelter, therapy dogs and pet dogs from both the current and our previous study [10] are provided in **Table 1.** for the available pre-treatment conditions.

**Table 1:** Mean±SE (min−max) change in bias scores of the three different populations of dogs for social interaction with the owner vs. stranger prior to the social susceptibility test. Data from Kis et al. 2022 [10] is included for comparison.

Pre-treatment	Former shelter s	Therapy dogs	Pet dogs	Pet dogs [10]
Owner	1.00±0.39 (-2–4)	1.61±0.51 (0–6)	2.25±0.47 (-1–6)	2.22±0.29 (0–5)
Stranger	1.14±0.57 (-1–5)	0.50±0.37 (-2–3)	1.29±0.37 (-2–4)	
Social ignoring			0.75±0.37 (-2–3)	0.79±0.33 (-2–4)
Social separation			0.68±0.41 (-3–4)	

## Discussion

Previous research has shown that social priming (i.e. social stimulation provided by the owner) has the potential to increase pet dogs’ social susceptibility in a similar way as would an intranasal oxytocin treatment [10]. It has been also shown that the oxytocin-increasing effect of social interactions depends on the relationship between the interacting partners [23]. Our results are in line with these former findings, as we found that pet dogs are most influenced in the social susceptibility task (as measured by fewer “bigger” choices in the human influence compared to baseline trials) if they had participated in a social pre-treatment by the owner. At the same time their behaviour following pre-treatment with a stranger does not differ from control conditions (Social ignoring, Social separation). This seemingly contradicts previous results suggesting that social contact (e.g. petting) by a stranger human would still increase dogs’ oxytocin levels [12]. However, given that the stranger pre-treatment is also not different from the owner pre-treatment condition, it seems that the difference in the oxytocin inducing effect of social interaction with the owner vs. stranger is of a small magnitude that needs a big sample size to be statistically detectable. We had expected for the social isolation condition (when the dog was left alone) to be more stressful and thus resulting in a differential behavioural change, compared to social ignoring (when the owner was present), however this was not the case. Since we did not measure cortisol (nor oxytocin) levels following the pre-treatments, we cannot directly compare the stress caused. We also cannot rule out a possible floor-effect, which could prevent dogs from going below a certain level of performance.

Dogs adopted from a shelter were involved to test the long-term effects stress caused by social deprivation [40, 41] on the way they are later affected by social stimulation. The results of this group were surprisingly similar to those found with pet dogs. This might show that despite a socially deprived early life history and some evidence of altered social behaviour of former shelter dogs [33, 42] their social susceptibility is similarly influenced by pre-treatment with social stimuli. For the former shelter dogs here we only focused on any potential differences between their behaviour following pre-treatment by a stranger versus their owner, thus we have no information on how this would compare to control pre-treatments. In the case of pet dogs, we found an indirect difference between owner and stranger pre-treatment. Although the difference between these two condition did not reach statistical significance, social susceptibility was significantly higher following the owner pre-treatment compared to the control (separation and ignoring) conditions, but not following the stranger pre-treatment. Former shelter dogs, on the other hand, gave the exact same response after both owner and stranger pre-treatments. There is some literature about how dogs living in shelters form attachment bonds with people [43], showing that dogs tend to respond differently in a strange situation test after just a few social interaction sessions with their “adopting owners” compared to when interacting with a completely unfamiliar human. This can be interpreted in a way that shelter dogs easily bond to their adopting owner, which would predict that former shelter dogs differentiate between their owners and a stranger human the same way as pet dogs with a normal life history would. On the other hand, the results of Gácsi et al. [43] can also be interpreted so that shelter dogs value all human social contact equally, and that the short social interaction used in the present study was enough for them to have the same effect as the pre-treatment with the owner. Visual inspection of the data seems to confirm the latter: the “change in bias” scores of former shelter dogs were at about the same level for both stranger and owner pre-treatments as were for pet dogs in the stranger, but not in the owner condition.

Therapy dogs are trained to engage in interactions with strangers with the aim of benefitting the human partner [34]. It has been shown that these dogs also experience therapeutic sessions as something positive in terms of behavioural and physiological measures of stress and relaxation [44]. Thus we expected that in the case of therapy dogs, interaction with a stranger would be something equally oxytocin-inducing as interaction with the owner. However, our findings suggest the opposite. Therapy dogs were clearly less susceptible to human influence following the stranger compared to the owner pre-treatment. Again, for this group we do not have data about control conditions (such as Social ignoring and Social separation). However, visual inspection of the results suggest, that while their “change-in-bias” score after the owner pre-treatment is comparable to that of pet dogs, following the stranger pre-treatment, the same score is lowest in this group and is at the same level as pet dogs following the ignoring and separation pre-treatment. This suggests that for therapy dogs who are often petted by strangers as part of their work, an interaction with a stranger does not have the same social value as interaction with their owner. Furthermore our results are potentially in line with those voices [45, 46] who raise welfare concerns about therapy dog work suggesting that forced social interactions might still be stressful, depending on the specifics of the experience dogs have.

We have to note that the social interaction used in the present experiment (following the protocol validated for increasing peripheral oxytocin [11]) included various elements, such as physical contact, eye-contact as well as dog-directed speech. Some previous research has already looked into how the different components of ostensive cues affect dogs behaviour [47, 48], however for the current study we do not have any information about how much each of these components contributed to the overall effect of social stimulation. A further limitation is, that comparison between dogs with different life history (pet dogs from a breeder, former shelter dogs, therapy dogs) is merely indirect in the present paper.

Taken together our results contribute to the extensive body of evidence, which is based on human data, suggesting that priming with affiliative stimuli can enhance prosocial predispositions [49, 50] and almost identically replicate previous findings on pet dogs [10] using the same social susceptibility paradigm. The novelty of the current findings lies in highlighting the difference in terms of behavioural reaction to social pre-treatment by the owner versus a stranger. This differential effect is further modulated by the previous life history of dogs, as evidenced by our results on therapy dogs and former shelter dogs. While we do have some indication [10, 11], that the behavioural changes observed are at least partly modulated by the neuro-hormone oxytocin, further research should look into the exact mechanisms modulating these processes.

## Supporting information

S1 Dataset (XLSX)
